# Acid and Alkali Taste Sensation

**DOI:** 10.3390/metabo13111131

**Published:** 2023-11-04

**Authors:** Prakash Pandey, Bhanu Shrestha, Youngseok Lee

**Affiliations:** Department of Bio and Fermentation Convergence Technology, Kookmin University, Seoul 02707, Republic of Korea; laliguranspandey@kookmin.ac.kr (P.P.); bhanu_totti7@kookmin.ac.kr (B.S.)

**Keywords:** taste, acid, alkali, OTOP1, Alkaliphile

## Abstract

Living organisms rely on pH levels for a multitude of crucial biological processes, such as the digestion of food and the facilitation of enzymatic reactions. Among these organisms, animals, including insects, possess specialized taste organs that enable them to discern between acidic and alkaline substances present in their food sources. This ability is vital, as the pH of these compounds directly influences both the nutritional value and the overall health impact of the ingested substances. In response to the various chemical properties of naturally occurring compounds, insects have evolved peripheral taste organs. These sensory structures play a pivotal role in identifying and distinguishing between nourishing and potentially harmful foods. In this concise review, we aim to provide an in-depth examination of the molecular mechanisms governing pH-dependent taste responses, encompassing both acidic and alkaline stimuli, within the peripheral taste organs of the fruit fly, *Drosophila melanogaster*, drawing insights from a comprehensive analysis of existing research articles.

## 1. Introduction

Sensory perception plays a vital role in the survival and well-being of animals, enabling them to navigate their environment and meet their fundamental requirements, such as securing nourishment, finding shelter, ensuring reproductive success, ensuring safety, and engaging in meaningful interactions with other members of their ecosystem. This complex procedure requires the synchronization of multiple separate sensory systems, each skilled in transmitting vital data to the brain for prompt analysis and reaction. Among these, mammals have evolved five primary sensory organs, namely the eye for vision, the ear for hearing, the tongue for taste, the nose for smell, and the skin for tactile perception, collectively facilitating the discernment of visual cues, auditory signals, flavors, scents, and tactile sensations. Much like other mammals, *Drosophila melanogaster* possesses distinct sensory organs that empower it to distinguish favorable from unfavorable environmental stimuli, enabling its survival. *Drosophila* exhibits a sophisticated sensory apparatus that aids in the recognition of external stimuli. Their remarkable compound eyes are particularly adept at facilitating various aspects of visual behavior, serving as a pivotal tool for environmental navigation and identification [1]. Furthermore, the species relies on the Johnston’s organ, situated within the antenna, which serves the dual purpose of detecting sounds and facilitating mechanosensation, thereby allowing fruit flies to respond to auditory and mechanical stimuli with precision [2,3,4,5]. In addition to these, *Drosophila* possesses a diverse array of olfactory organs, each specialized in the detection and processing of a wide range of odors [6]. These olfactory receptors play a critical role in the fly’s ability to identify and respond to specific volatile chemical cues within their surroundings. Moreover, the gustatory organs of the fruit fly enable the perception of taste, allowing them to differentiate between various food sources and potentially harmful substances [7,8,9,10,11,12]. This intricate sensory system collectively equips *Drosophila* with the tools necessary for efficient perception and response to the external environment. Within the adult body of *Drosophila*, hair-like projections develop into taste organs in various locations, including the proboscis, legs, wing margins, and ovipositor [13,14,15,16,17,18,19]. Of these, the tip of the proboscis comprises the bifurcate labellum, which plays a particularly crucial role in detecting taste by coming into contact with food or chemical compounds. The labellum of *Drosophila* contains 31 taste sensilla on each side, arranged symmetrically. These sensilla are instrumental in chemosensations, especially concerning non-volatile compounds. There are three distinct types of sensilla in the labellum, short (S-type), intermediate (I-type), and long (L-type), differentiated by their size [16,20,21]. Each sensillum is innervated by two to four gustatory receptor neurons (GRNs), one mechanosensory neuron, and three supporting cells, with their signals projected to the subesophageal zone (SEZ) of the brain, the region responsible for taste sensation [13,22,23,24]. Additionally, taste sensation is also mediated by the hairless labellar taste peg situated between pseudotracheal rows, which is innervated by one chemosensory neuron and one mechanosensory neuron [25]. It is believed that taste pegs can only detect food when the flies open their labial palps. Furthermore, the adult fly pharynx, which acts as an internal molecular sensor, contains three different hairless internal taste organs: the dorsal cibarial sense organ (DCSO), the ventral cibarial sense organ (VCSO), and the labral sense organ (LSO) [26].

More precisely, the presence of specific molecular components like gustatory receptors (GRs) [7,27], ionotropic receptors (IRs) [28,29], pickpocket (PPK) ion channels [30,31,32], and transient receptor potential (TRP) ion channels [33,34] acts as mediators between external chemical cues and the fruit fly’s brain. These components play a crucial role in transducing chemical information into neural signals. The GRNs present in sensilla are sensitive to different types of chemical stimuli entering via pores at the tip of the sensilla. For example, the S-type sensilla contains four different GRNs, each responding to bitter or aversive compounds, sweet tastes, water, or specific minerals such as Na^+^ and Ca^2+^ [13,18,19,32]. In contrast, the L-type sensilla possesses four sets of GRNs that are sensitive to sweet tastes, water, low concentrations of salt, and others which have not yet been identified [19,35,36,37,38]. Similarly, the I-type sensilla are equipped with two sets of GRNs responding to sweet and bitter compounds, including both low and high concentrations of salt [39,40,41]. In this intricate web of molecular and sensory interactions, *Drosophila*’s taste organs play a pivotal role in helping the fly navigate its environment, ensuring its sustenance and survival. These sensory mechanisms provide a fascinating window into the broader world of biological systems and their adaptation to complex ecological challenges.

Taste preferences in *Drosophila* also have profound ecological implications. They guide foraging behavior, influence food source selection, impact breeding site choices, and even contribute to competition and niche partitioning among different *Drosophila* populations. *Drosophila*’s taste neurons and receptors assess food sources, shaping their preferences and guiding foraging. Favorable food sources lead to concentrated fly populations, impacting resource distribution. *Drosophila* can also act as pollinators, spreading pollen from one plant to another as they feed on nectar, affecting plant ecology. Taste cues guide females to identify and select appropriate substrates for oviposition. Consequently, taste preferences influence the distribution of *Drosophila* larvae within their environment, impacting their development and survival. Diverse taste perception facilitates niche partitioning, enabling the coexistence of multiple *Drosophila* species by exploiting different food sources and reducing competition. Understanding these taste preferences is essential for unraveling the intricate ecological dynamics and interactions between these fruit flies and their environment.

Research on the perception of attractive tastes, like sugar, in *Drosophila* has predominantly concentrated on measuring feeding initiation and food consumption. Nevertheless, it is vital to recognize that attractive compounds have a broader impact on the behavior of these fruit flies in relation to feeding. When *Drosophila* detects the taste of appealing compound, they display a strong inclination to congregate in those areas, and their positional behavior is significantly biased towards attractive compound-laden food sources [42,43,44,45]. The underlying reason for this preference is multifaceted. Firstly, some attractive tastes like sugar serve as a rapid source of calories for fruit flies, allowing them to sustain their energetic needs and engage in activities like flying. Furthermore, their presence indicates a level of nutritive value in the food source, prompting their neural pathways to bypass the protein circuit in favor of the sugar-rich option. This phenomenon extends to carbohydrate-containing foods, as flies are naturally drawn to sweet-tasting substances due to the promise of readily available energy. Conversely, *Drosophila* exhibits a marked aversion to bitter tastes, which often serve as a warning signal for the presence of potentially harmful or toxic substances [27,46,47,48,49,50]. This aversion to bitterness is not a mere preference, but an essential survival instinct. 

*Drosophila*’s food preferences go beyond taste alone, influenced by nutritional needs, genetic variation, environmental conditions, and learned behavior. While taste guides their initial preferences, nutritional requirements can override taste, and genetic diversity affects their perception of specific compounds [51,52]. Environmental factors, like food availability and competition, also play a significant role. *Drosophila* can learn from experience, allowing them to adapt and make optimal food choices. These multifaceted factors ensure their adaptability and reproductive success in diverse environments.

**Figure 1:**
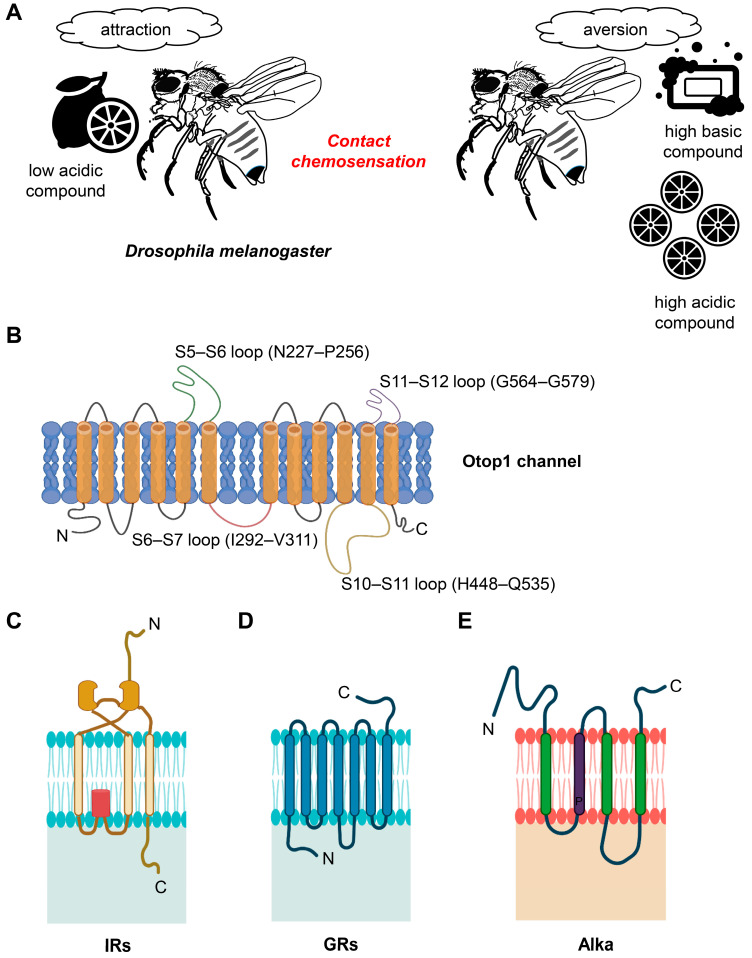
Behavior of acid and basic compounds and structure of OTOP1, GRs, IRs, and Alka. (**A**) Representation of *D. melanogaster*’s sensitivity to pH Levels. *D. melanogaster* is attracted to compounds with low acidity (low pH) while displaying aversion to highly acidic and highly basic compounds (high pH). (**B**) Human Otop1 channel membrane configuration. The human Otop1 channel is thought to have a membrane-spanning structure, with the N domain encompassing transmembrane segments S1 through S6, the C domain including S7 to S12, interconnected loops between these segments, and intracellular termini. (**C**) Architectural features of GRs. GRs possess a unique architectural composition characterized by seven transmembrane domains (TMDs), with their C-terminal regions located externally, distinguishing them from typical G-protein coupled receptors. (**D**) Topology of IRs. IRs exhibit a three-TMD structure, including a pore region, which shares structural similarities with mammalian glutamate receptors. (**E**) Proposed structural arrangement of alka. Alka is anticipated to have a structural configuration that includes a persistent proline residue (P) within the TM2 segment.

Sourness, characterized by low pH or acidity, represents a fundamental taste sensation [53]. It is universally appealing in moderate concentrations, yet becomes unappealing at higher levels, across both vertebrate and invertebrate species [54,55,56,57]. Conversely, foods with high pH or alkaline properties generally lack appeal [58,59,60]. Nevertheless, in mildly alkaline conditions, food with compounds like ammonia and certain amines is preferred, influenced by the intricacies of the sensory system [61,62] (Figure 1A). Notably, the attraction to mildly basic food is believed to be linked to the presence of low concentrations of salt [58]. Commonly, acidic components are found in raw fruits and spoiled food items, adding to the significance of detecting sourness as a warning sign. Conversely, basic or alkaline taste is triggered in certain vegetables, legumes, nuts, and other items due to their elevated pH levels. Current research across various species, both vertebrate and invertebrate, has substantiated that basic taste also qualifies as one of the fundamental taste qualities [58,60,63,64,65].

The pH levels in food have a direct impact on the physiology of living organisms, and can potentially disrupt their biological systems, sometimes leading to life-threatening consequences. For instance, the consumption of high-pH or alkaline foods can disrupt the acid–base balance in an organism, resulting in a condition known as alkalosis, which can be perilous to health [66,67]. Likewise, the accumulation of CO_2_ in the atmosphere increases the likelihood of its dissolution in water, leading to a reduction in the water’s pH level [68]. This shift in pH can pose a significant threat to the well-being of aquatic organisms. Given these critical physiological consequences, it becomes imperative for living organisms to distinguish between available food sources based on their pH levels. Extensive research efforts have been devoted to unraveling the mechanisms behind sour and basic tastes, with a focus on identifying the receptors and transduction pathways responsible for these sensory perceptions. In this review, we delve into the existing body of literature to provide insights into the mechanisms underlying acid and alkaline taste sensations in *Drosophila*, shedding light on how this tiny insect processes and responds to different pH levels in its environment.

## 2. pH-Dependent Taste Quality

Sour taste, a naturally occurring taste quality, is primarily determined by the presence of acidic compounds in food. The Rutaceae family, known for its sour-tasting fruits, owes this characteristic to a high concentration of citric acid [69]. Additionally, a wide array of foods, including fermented Korean kimchi, baked goods, beverages, confections, gelatin desserts, jams, jellies, dairy products, processed meats, fats, and oils, incorporate various acids to enhance their flavors [70]. Numerous acidulants exhibit the ability to chelate trace metal ions, and they often collaborate effectively with antioxidants [70]. Moreover, food acids find diverse applications in culinary processes, serving as curing agents, modifying viscosity and melting characteristics, thwarting nonenzymatic browning, and acting as effective antioxidants to prevent oxidation [71].

Acids can be categorized into two groups: inorganic and organic acids. Inorganic acids such as hydrochloric acid, nitric acid, and sulfuric acid, as well as organic acids like lactic acid, citric acid, malic acid, and acetic acid, contribute to the perception of sour taste [72]. The sourness in strong inorganic acids is driven by the presence of protons (H^+^ ions), with the intensity of sourness directly linked to the number of dissociated proton ions [73,74]. Organic acids contain at least one carboxyl (-COOH) functional group, with the dissociation of the hydrogen from this group into an H^+^ ion being influenced by the acid’s strength. The concentration of this ion, along with the concentration of protonated organic acids, constitutes the key source of sour taste [75,76,77]. An intriguing aspect of sourness is that weak acids at the same pH level can be perceived as more sour than strong acids, highlighting the role of undissociated weak acids in evoking a sour taste [78].

On the contrary, an alkaline taste arises from chemical compounds that dissociate into hydroxyl groups (OH^−^) and possess a pH value higher than 7 in food [63]. Substances such as sodium hydroxide (NaOH) and carbonates (CO_3_^2−^) naturally occur in food and water [58]. They are also added to food during industrial production as antimicrobial food additives and acidity control agents. Naturally, compounds containing an amino (-NH_2_) group covalently attached to a carbon backbone exhibit basic properties and contribute to higher pH levels. Aqueous ammonia is a prime example of an alkaline substance. Additionally, alkaloids, which are basic compounds, are aversive to insects [79,80,81]. Notably, fundamental environmental elements such as soil, which provide a nurturing ground for various plant species, often exhibit elevated pH levels.

The ability to perceive and react to the sourness of acidic compounds and the alkaline nature of basic substances is essential for the survival and well-being of insects. These taste qualities play a critical role in helping organisms make informed dietary choices and avoid potentially harmful or toxic substances.

## 3. Molecular Mechanism of Acid Sensation

The perception of sour taste, resulting from the presence of acidic compounds in food, has long intrigued scientists, leading to a gradual understanding of how the peripheral taste system in animals detects this taste, alongside other fundamental tastes. Due to variations in the chemical sensitivity of taste receptors among different animal species, identifying a single universal channel responsible for sour taste detection proved challenging [82]. However, researchers persisted and studied a wide range of species, including *Drosophila* (Table 1) and vertebrates (Table 2), to unravel the cellular mechanisms behind sour taste transduction.

A different study proposed a widely accepted theory that sour taste perception involves acid molecules breaching cell membranes to release hydrogen ions [91,92], activating sour taste receptors in specialized taste receptor cells (TRCs) on the tongue [93,94]. Throughout evolution, animals have developed a diverse array of proton-activated channels and G-protein-coupled receptors (GPCRs) to distinguish acidic pH levels. Various potential receptors were considered, including the acid-sensing ion channel 2 (ASIC2) [95] and hyperpolarization-activated cyclic nucleotide gated (HCN 1 and HCN 4) channels [96,97], but their roles were inconclusive [98,99]. Polycystic kidney disease 2-like 1 (PKD2L1) was initially thought to be a sour taste receptor [100,101], but further studies found its role to be modest [102,103]. Ultimately, Otopetrin 1 (OTOP1) emerged as a pivotal sensor in sour taste perception, marking a significant milestone in our understanding of animal taste sensation [87,88,89] (Figure 1B). Further, researchers discovered that lowering extracellular pH increases inward current in Type III taste receptor cells, indicating their role in detecting sourness [104]. OTOP1 is identified as essential in sour taste transduction, with experiments confirming pH-dependent currents [87,104]. Cryo-EM analysis revealed OTOP1’s dimeric structure with 12 transmembrane helices [105]. The mechanism of OTOP1 gating for proton permeation remains unknown, warranting future research. Fruit flies possess Otopetrin-like a (Otop-La) as a functional ortholog of OTOP1 [55,56]. Otopetrins are a family of proteins that have been implicated in sour taste perception in various organisms, including mammals and fruit flies [55,56]. This protein is part of the molecular machinery that allows fruit flies to perceive and respond to sour tastes, ensuring their ability to make informed dietary choices. Understanding the presence of Otop-La in fruit flies adds to our knowledge of the molecular mechanisms underlying sour taste perception in insects, contributing to our broader understanding of taste sensation across different species.

Behavioral analysis in fruit flies demonstrated a bidirectional response to diets with varying acid concentrations, showing attraction to low-acid and aversion to highly acidic food [54,57,106]. Electrophysiological analyses revealed that L-type sensilla were responsible for the attraction to acid-containing foods, while S-type sensilla were involved in exhibiting aversion [54,57,106]. Nevertheless, this outcome does not imply that L-type sensilla solely responds to attractive compounds such as low pH compound, while S-type sensilla is solely responsible for exhibiting neuronal response to aversive compounds like high pH compound. Researchers showed that IR7c-expressing GRNs, which are required to sense high concentrations of salt (aversive nature), are expressed in most of the L-type sensilla and few S-type sensilla [37]. This suggests that even L-type sensilla could serve as a neuronal responder, contributing to the detection of a compound known to induce aversive behavior. In addition, sensilla S4 and S8, akin to L-type sensilla, demonstrated heightened neuronal responses to appealing concentrations of salt, underscoring the likelihood that S-type sensilla also exhibited increased neuronal activity in response to enticing compounds [107]. Thus, different sensilla exhibit different neuronal response for various tastants, including acidic compounds, and the exact connectome between S-type and L-type sensilla is yet to be established. 

Two recent studies by Mi et al., 2021 and Ganguly et al., 2021 shed light on the OtopLA channel’s role in *Drosophila*’s gustatory system [56,56]. Mi et al. demonstrated the distinct nature of OtopLA-expressing GRNs, elucidating their contribution to attractive responses at lower acid concentrations [55]. Despite some observed overlap with sweet-sensing GRNs, the SEZ projection region of the *otopla*-expressing GRNs appeared to be segregated from those associated with sweet-, bitter-, and salt-sensing GRNs, thus revealing a previously unrecognized subset of GRNs [55]. In contrast, Ganguly et al. found an approximately 50% expression of *otopla* across various GRNs, including *Gr64f*-positive (sweet), *Gr66a*-positive (bitter), *ppk28*-positive (water), and *ppk23*-positive (cation) GRNs [56]. Moreover, their research emphasized the role of *otopla* in both attractive and aversive acidic stimuli, a finding reinforced by their impactful recovery experiments. Diverging from Mi et al.’s study, Ganguly et al. conducted meticulous cell-type-specific rescue experiments, revealing that OtopLA functions in *ppk23*-positive GRNs for repulsion and *ppk28*-positive sensing GRNs for attraction to high and low levels of protons present in food, respectively [56]. In summary, both studies collectively underscore the critical role of OtopLA in the complex realm of gustation, significantly expanding our understanding of the fundamental principles governing sensory perception. These findings not only enhance our comprehension of the intricacies of the *Drosophila* gustatory system, but also provide valuable insights into how both humans and animals assign emotional responses to various stimuli based on their intensity levels.

Further research showed that although a small proportion of *otopla*-expressing GRNs appeared to overlap with sweet GRNs, most *otopla*-expressing GRNs were distinct from those responding to sweet, bitter, and salty stimuli in the proboscis [55]. These GRNs mainly represented a distinct group found in L-type sensilla, responsible for detecting low acid (pH) foods. In addition to this, another group of researchers found that the Otop-La channel plays a pivotal role in orchestrating responses to both appealing and repelling stimuli related to acidic foods [56]. This channel serves as a crucial mediator for attraction to mild acidic taste and aversion to highly acidic tastes. Intriguingly, humans share a similar pattern of perception, as we tend to find low concentrations of acids appetizing while reacting negatively to higher concentrations. The researcher’s exploration of the cellular and molecular mechanisms underlying acid sensing, specifically within the Otop-La channel, provides a valuable foundation for exploring into the intricate processes that determine how animals ascribe positive or negative values to stimuli that primarily differ in their intensity levels. This insight promises clarification on the fundamental principles governing sensory perception and the assignment of emotional responses to various sensory experiences.

Additionally, an ionotropic receptor member, *Ir7a*, expressed in some bitter GRNs, was identified as a sensor for acetic acids [54]. The narrowly tuned IR7a, required to stop *Drosophila* from feeding on foods laced with acetic acid, does not affect the rejection of foods containing HCl or any other carboxylic acids examined. IR7a can detect excessive acid concentrations with low pH alongside acetate anion. Attractive carboxylic acids containing an α-hydroxyl group, such as lactic acid and glycolic acid, were mediated by broadly tuned receptors like IR25a and IR76b [83,84,108]. Interestingly, sweet gustatory receptors, such as GR5a, GR61a, and GR64a–GR64f, played a role in attenuating the attractive responses to these α-hydroxy acids [83,84,108]. Lactic acid, an energizing and appetizing stimulant, activates the specific mechanical pathways of sweet GRNs. The onset response, which triggers feeding initiation by causing the proboscis extension, is predominantly mediated by the ionotropic receptor. In contrast, the off response to low pH is mediated by sweet gustatory receptors, which predominantly affects food intake [83]. The sensation of vitamin C (ascorbic acid), which enhances hunger resistance and triggers egg laying in *Drosophila melanogaster*, involved sugar-sensing GRs and IRs [45] (Figure 1C,D). Long-chain fatty acids (FAs), which are slightly acidic but energy-rich nutrients, could also elicit taste sensations. In *Drosophila*, the *Gr64* cluster comprises six tandem GR genes (*Gr64a-Gr64f*) that play an essential role in the detection of these fatty acids [109]. Both electrophysiological and behavioral data point to GR64e as a crucial receptor that contributes significantly to FA sensation [109]. Furthermore, the taste perception of FAs involves sweet-sensing GRNs, and the broadly expressed receptors IR25a and IR76b, along with IR56d, are essential for detecting these compounds [110]. Notably, IR56d plays a specific role in responding to medium-chain fatty acids, such as 6C, 7C, and 8C fatty acids [111]. Phospholipase C signaling in the sweet-sensing neurons of *Drosophila* is a well-preserved molecular signaling pathway that is responsible for eliciting an attractive response to fatty acids [112]. When it comes to hexanoic acid-induced attractive behavioral responses, they reach their peak at a 1% concentration but diminish at higher concentrations. PER assay analysis demonstrates that this phenomenon is mediated by the independent activation of bitter GRNs by IR25a and IR76b [85]. Furthermore, GR64d and IR56d are necessary via sweet-sensing GRNs in L-type sensilla to recognize an alluring hexanoic acid concentration of 0.1%. On the other hand, three bitter GRs (GR32a, GR33a, and GR66a) are primarily responsible for inducing aversion to a 1% hexanoic acid concentration, which is mediated by bitter-sensing GRNs in S-type sensilla [85]. Gustatory behaviors of three *Drosophila* species, *D. sechellia*, *D. simulans*, and *D. melanogaster* revealed substantial evolutionary modifications in the behavioral responses of the three species to noni FAs [86]. Studies examining taste sensory responses to noni FAs indicate notable distinctions in the mechanisms of FA taste recognition between the appetitive sweet-sensing and deterrent bitter-sensing taste neurons in *D. sechellia*, in comparison to the generalists, which are linked to the shift in its feeding preference for noni. An analysis of chemoreceptor mutants in *D. melanogaster* suggests that multiple genetic alterations contribute to the evolution of gustatory behavior in *D. sechellia*. Moreover, receptors in various parts of the body, beyond the proboscis, also contribute to acid taste sensation. The majority of tarsal sensilla contain a sour GRN specifically activated by carboxylic and mineral acids but not by sweet, bitter, or salty chemicals [104]. These sour GRNs also prominently express two IRs, IR25a and IR76b, which are essential for acid detection. 

Carboxylic acids can suppress feeding by activating bitter-sensing GRNs and inhibiting sugar responses in sweet-sensing GRNs [106]. A recent study focused on citric acid and L-type sensilla (L7), housing sweet-sensing GRNs alone, confirming its concentration-dependent suppression of sugar responses [56]. However, hydrochloric acid (HCl) at a pH of 2.0 did not exhibit the same effect on sweet-sensing GRNs, highlighting distinct acid–taste interactions. This curious deviation from the pattern observed with carboxylic acids suggests that not all acids act in a uniform manner in the gustatory system. Also, the taste system’s adaptability allows animals to respond differently to the same substance based on their immediate requirements. Research involving behavioral and neural reactions to acetic acid has unveiled a fascinating phenomenon: fruit flies adjust their response to this stimulus through a hunger-dependent switch [113]. When flies are well-fed, they exhibit a taste aversion to acetic acid. In contrast, starved flies display a robust appetitive response to the same substance. Hunger plays a pivotal role in this transformation of behavior by boosting the appetitive sugar pathway while simultaneously inhibiting the aversive bitter pathway. This study demonstrates how a single taste compound can elicit contradictory behaviors by activating distinct taste pathways, which are modulated by the internal state of the organism. The implications of this research extend beyond fruit flies, as it points towards a broader understanding of how the brain integrates taste information and hunger signals to regulate food consumption. Such insights may hold the key to addressing and treating obesity in the future.

## 4. Alkali Detection in Taste

Just as low pH (acidity) produces a gustatory sensation known as sourness, it is logical to hypothesize that high pH (alkalinity) might also elicit a distinct taste sensation. Early scientific inquiries suggested that alkali compounds could evoke specific types of tastes, such as sour and sweet, by exciting taste receptors [114,115]. For example, studies dating back to 1948 observed that the tip of the human tongue had a higher sensitivity to sodium hydroxide solution than other regions, providing early evidence for an alkaline taste sensation [63]. Electrophysiological recordings of taste nerves in cats also indicated that a subset of these nerves could be activated by high pH, suggesting the existence of an alkaline taste [64]. Trigeminal neurons, a type of sensory neurons, respond to various external alkaline pH levels [64]. Among the TRP channels, TRPV1 and TRPA1 are known to be activated by intracellular alkalization but not by exposure to external alkaline pH alone [116,117]. It is important to note that these channels can also be activated by acidic pH levels. 

In the context of studying *C. elegans*, TMC-1 plays a role in mediating alkaline sensation through ASH nociceptive neurons [118]. TMC-1 is one of the two TMC family genes found in *C. elegans* and is believed to encode a sodium-sensitive channel that is required for salt chemosensation and food signaling. When exposed to alkaline pH, ASH neurons exhibit an inward current that is primarily dependent on TMC-1 and only secondarily dependent on the TRPV channel OSM-9. While OSM-9/TRPV is sensitive to both acidic and basic pH, TMC-1 displays specificity towards alkaline conditions. It is essential not only for the electrical current in ASH neurons, but also for the behavioral response triggered by alkaline pH, whereas it is not involved in the response to acidic pH [118].

Research on carabid beetles and ground beetles demonstrated that these insects exhibit a strong aversion to alkaline conditions, particularly in relation to their habitats and food sources [119]. These studies suggested that insects have taste receptors that can detect high pH levels and influence their feeding behavior. Furthermore, this study holds ecological significance, as the aversion to alkaline conditions influences habitat selection, affects soil quality and vegetation composition, impacts community dynamics, and holds implications for conservation and land management practices across diverse ecosystems. It highlights the intricate web of interactions and dependencies that characterize ecological systems. In the context of *Drosophila* research, it was discovered that alkaline substances with a high pH could indeed generate a gustatory sensation, implying the presence of a separate channel in the taste organ dedicated to alkaline taste perception. Further investigation revealed the importance of a chloride channel called Alkaliphile (Alka) in fruit flies’ adverse taste reactions to basic foods (Figure 1E). Alka selectively creates a high pH-gated chloride channel in specific GRNs, enabling the detection of alkaline taste [58]. Additionally, it was found that high pH conditions suppressed the sugar-based neuronal response triggered by sweet-sensing GRNs, supporting the notion that the detection of high pH involves dual mechanisms: the activation of certain bitter-sensing GRNs and the inhibition of sweet-sensing GRNs. This discovery in fruit flies has paved the way for future studies exploring how other organisms’ peripheral taste organs perceive alkaline tastes at the molecular level.

Additionally, research in vertebrates, specifically zebrafish, revealed the involvement of OTOP1 in basic taste sensation [60]. OTOP1 was found to mediate proton inflow and efflux in response to extracellular acid and base stimulation. Notably, the mutation of specific domains within OTOP1 affected its affinity for alkali compounds without impacting its response to acidic stimuli, highlighting the distinction between acid and alkali activation [60]. The mouse study demonstrated that OTOP1 functions as a sensor for ammonium chloride (NH_4_Cl) [90]. Taste responses to NH_4_Cl, as measured from isolated Type III TRCs or gustatory nerves, were significantly reduced or completely absent in an *Otop1^−/−^* mouse. Recent research into the gustatory system using *Drosophila* and *Aedes aegypti* has found that the perception of sweetness and saltiness can be inhibited as the basicity (pH) of a tastant solution increases, particularly with the addition of ammonium hydroxide (NH_4_OH) [59]. This intriguing discovery challenges our understanding of taste, and holds potential implications for industries like food and beverage industries, as it suggests that altering basicity could influence flavor perception and innovation.

In the context of acidic food conditions, an intriguing research avenue emerges with adaptability based on the changing condition of internal body state [113]. Exploring the impact of alkaline presence on internal states and consequent behavioral changes presents an exciting opportunity for investigation. Delving deeper, it becomes fascinating to examine how these state-dependent alterations influence the functioning of sensory neurons, particularly in the sugar and bitter circuits downstream. Additionally, a compelling aspect of this research could involve studying the switch in response to hunger levels. This may explain how organisms adapt to alkaline food, which could be considered a potentially aversive compound, yet may offer nutritional benefits in extreme conditions. The exploration of alkaline taste, like sour and other taste modalities, continues to unravel the intricacies of the gustatory system and how organisms perceive and respond to a wide range of chemical stimuli in their environment.

## 5. Conclusions and Future Perspectives

The sense of taste in insects is a crucial survival mechanism that allows them to distinguish between nutritious, safe foods and potentially harmful substances in their environment. Acidic and alkaline compounds naturally occur in various foods and environmental factors, and their presence directly impacts the health and well-being of living organisms. While there has been significant research into the molecular receptors responsible for detecting acidic and sour tastes, the sensation of alkaline or basic tastes remains a relatively understudied area. Understanding how organisms perceive and respond to alkaline compounds is an important avenue of research, as it can shed light on their dietary choices and survival strategies.

In the case of *Drosophila* and other organisms, the role of IRs in the sensation of acidic tastes is still not fully understood. Future studies will likely elucidate the mechanisms through which Otop-La and IRs concurrently mediate the perception of acidic taste. Likewise, investigating whether and how these receptors function in response to alkalic compounds can provide valuable insights into the molecular mechanisms underlying alkaline taste perception. Furthermore, exploring the effects of basic compounds on the metabolism and physiology of both mammals and insects represents another intriguing area of study. Understanding how alkaline substances may impact metabolic processes, nutrient absorption, or other physiological functions can have implications for both basic science and potential applications in fields such as agriculture or pest control.

While our discussion has primarily centered on peripheral sensation, it is worth noting that recent research has increasingly emphasized the role of internal sensors in various organisms, including *Drosophila*. These internal sensors enable the detection of nutrients such as sugars and amino acids, not only in the peripheral taste receptors but also in the pharynx, gut, and even within the central nervous system in the brain. As ongoing studies continue to explore these internal sensory mechanisms, we can expect to uncover answers to many intriguing questions related to how organism sense and respond to internal chemical cues. This emerging field holds significant promise and has the potential to yield valuable insights into the complex interplay between sensory perception and internal physiological processes. It indeed represents an area of high impact and importance for future research and understanding.

Studying multisensory interactions in the simple insect model, *D. melanogaster*, holds great potential for advancing our understanding of sensory processing, and can pave the way for the development of environmentally friendly insecticides in agriculture. Taste perception plays a crucial role in the effective development of insecticides to combat harmful pests. Previous research has demonstrated that certain insecticides function as both antifeedants for adult insects and as larvicides [120]. Additionally, when lower doses of antifeedant compounds are combined, they synergistically enhance the antifeedant effect [120]. This discovery offers a promising path toward creating safer and more efficient antifeedants. Practical experiments should be conducted to evaluate the impact of this combined treatment in real-world settings, such as fields, crop storage, and insect vector control. Higher pH or concentrated compounds might have a similar effect. Therefore, exploring the role of repelling sour or alkaline sources in eradicating harmful insect vectors or pests could be advantageous in improving human health by reducing reliance on synthetic insecticides. Furthermore, leveraging basic research on the fruit fly can help us unravel the taste system in higher animals, discover new artificial additives or drugs that have higher impact, and improve quality in the food industry. Taste perception research has the potential to revolutionize both agriculture and the food industry.

In summary, the study of taste sensation, particularly in relation to alkaline compounds, holds the potential for significant discoveries that can advance our understanding of the gustatory system, dietary choices, and survival strategies of animals. It also has broader implications for various scientific disciplines and practical applications.

## Figures and Tables

**Table 1:** Receptors required for detecting acidic and basic compounds in *Drosophila melanogaster*.

Category	Stimuli	Receptors	Reference
Acid (attractive)	hydrochloric acid (low) and other organic acids	Otop-La	[55,56]
	lactic acid, citric acid	IR25a, IR76b, GR5a, GR61a, GR64c, GR64d, and GR64e	[83,84]
	glycolic acid	IR25a, IR76b, GR5a, GR61a, GR64c, and GR64d	[45,84]
	vitamin C	IR25a, IR76b, GR5a, GR61a, GR64b, GR64c, and GR64e	[45]
	hexanoic acid (low), octanoic acid, oleic acid, and linoleic acid	GR64d, GR64e, GR64af, IR25a, IR56d, and IR76b	[85,86,87,88]
Acid (aversive)	HCl (high) and other organic acids	Otop-La	[55,56]
	acetic acid	IR7a	[54]
	hexanoic acid (high), octanoic acid, and decanoic acid	GR32a, GR33a, GR66a, IR47a, and IR76b	[85,86]
Alkali	NaOH and Na_2_CO_3_	Alka	[58]

**Table 2:** Receptors required for detecting acidic and basic compounds in vertebrates.

Category	Stimuli	Receptors/Cell	Reference
Acid	HCl, citric acid, and tartaric acid	OTOP1/Type III TRCs	[87,88,89]
Alkali	NaOH and NH_4_Cl	OTOP1	[60,90]
